# Integrated chemical and genetic authentication of Polygoni Multiflori Caulis: Refuting diameter restrictions and exposing market adulteration

**DOI:** 10.7717/peerj.21561

**Published:** 2026-08-03

**Authors:** Ngoc-Phuoc Ngo, Meng-Shiou Lee, PeiJung Chiang, Jing-Yao Huang, Ho-Ling Hung, Kun-Chang Wu

**Affiliations:** 1 School of Pharmacy, College of Pharmacy, China Medical University, Taichung, Taiwan; 2 Institute of Pharmaceutical Education and Research, Binh Duong University, Ho Chi Minh, Vietnam; 3 Department of Chinese Pharmaceutical Sciences and Chinese Medicine Resources, College of Chinese Medicine, China Medical University, Taichung, Taiwan; 4 Department of Traditional Chinese Medicine, Taichung Veterans General Hospital, Taichung, Taiwan; 5 Graduate Institute of Chinese Medicine, School of Chinese Medicine, China Medical University, Taichung, Taiwan; 6 He Yi Trading Co., Ltd., Kaohsiung, Taiwan; 7 Department of Pharmacy, China Medical University Hsinchu Hospital, Hsinchu, Taiwan

**Keywords:** Polygoni Multiflori Caulis, *Pleuropterus multiflorus*, ITS2 sequencing, HPLCfingerprinting, SNP markers, Quality control, Adulteration, *Polygonum multiflorum*, *Polygonum multiflorum* var. *hypoleucum*, *Pleuropterus angulatus*

## Abstract

**Background:**

Polygoni Multiflori Caulis (PMC), the dried stem of *Pleuropterus multiflorus* (Thunb.) Turcz. ex Nakai (PM), is a fundamental traditional Chinese medicine utilized for treating sleep disorders. Despite its medicinal importance, species confusion exists due to the taxonomic synonymy of *Polygonum multiflorum* var. *hypoleucum* (PMH) and *Pleuropterus angulatus* (PA) with PM. Furthermore, conventional quality standards often restrict PMC collection to specific stem diameters. This study evaluatedthe chemical and genetic diversity of PMC and its related taxa and assessed the authenticity of commercial PMC products.

**Methods:**

Authenticated samples of PM, PMH, and PA were analyzed using high-performance liquid chromatography (HPLC) to quantify three key markers, including 2,3,5,4′-tetrahydroxystilbene-2-*O*-*β*-D-glucoside (THSG), emodin, and physcion. The ITS2 region was sequenced to evaluate genetic diversity and identify single nucleotide polymorphisms (SNPs). We examined the impact of stem diameter (2.0 to 25.1 mm) on constituent levels in PM and surveyed eleven commercial PMC samples from Taiwanese markets.

**Results:**

Chemical analysis revealed that PMC samples across the entire diameter range (2.0 to 25.1 mm) consistently fulfilled the regulatory standards of the Taiwan Herbal Pharmacopoeia (THP), Chinese Pharmacopoeia (CP), and Hong Kong Chinese Materia Medica Standards (HKCMMS), effectively challenging the previously recommended 4 to 7 mm limit. In contrast, authenticated PMH samples lacked both the characteristic marker THSG and the enlarged tuberous roots typical of PM, and PA stem samples exhibited variable chemical phenotypes, with only a small subset meeting official standards. ITS2 sequencing successfully resolved the three taxa by identifying 13 SNPs and 16 distinct SNP genotypes. Notably, the market survey revealed that 82% of commercial samples were substituted with PA, of which 72% contained undetectable levels of the required analytes.

**Conclusions:**

The findings demonstrate that PMH and PA represent distinct chemotypic lineages and should not be considered interchangeable botanical sources for PMC. The integration of HPLC quantification and ITS2 SNP barcoding provides a robust, multifaceted framework for quality control. Furthermore, our findings suggest that stems across the 2.0–25.1 mm diameter range can meet current pharmacopoeial requirements, providing preliminary evidence that the traditional 4–7 mm restriction may be unnecessarily narrow.

## Introduction

Polygoni Multiflori Caulis (PMC), known as Shou-Wu-Teng or Ye-Jiao-Teng, is derived from the dried stems of *Pleuropterus multiflorus* (Thunb.) Turcz. ex Nakai (syn. *Polygonum multiflorum* Thunb., hereafter PM), and is a cornerstone of traditional Chinese medicine (TCM). It is clinically indispensable for treating sleep disorders, rheumatic arthralgia, and skin pruritus (Chinese Pharmacopoeia Commission, 2025; Editorial Committee of the Hong Kong Chinese Materia Medica Standards, 2018; Editorial Committee of the Taiwan Herbal Pharmacopoeia, 2021; Plants of the World Online, 2026). In Taiwan, PMC ranks as one of the most frequently prescribed single herbs for patients suffering from insomnia and major depressive disorders (Chen et al., 2021; Chen et al., 2015). Its wide-ranging pharmacological properties, such as antioxidant, anti-obesity, antidepressant, and anti-inflammatory effects, have been thoroughly validated (Li et al., 2022; Shi et al., 2009; Shi & Xiao, 2009; Xiong et al., 2023).

The therapeutic efficacy of PMC is attributed to its diverse phytochemical profile, characterized by stilbene glycosides (notably 2,3,5,4′-tetrahydroxystilbene-2-*O*-*β*-D-glucoside, THSG), anthraquinones (emodin and physcion), and flavonoids (Liang, Tian & Ma, 2009). To ensure clinical safety and efficacy, the Taiwan Herbal Pharmacopoeia (THP) and the Chinese Pharmacopoeia (CP) mandate a minimum THSG content of 0.20%, while the Hong Kong Chinese Materia Medica Standard (HKCMMS) requires total anthraquinones to reach 0.034% (Chinese Pharmacopoeia Commission, 2025; Editorial Committee of the Hong Kong Chinese Materia Medica Standards, 2018; Editorial Committee of the Taiwan Herbal Pharmacopoeia, 2021).

However, quality control in the commercial market remains challenging due to two critical issues: physical standardization and botanical authenticity. First, although the THP specifies an ideal stem diameter of 4–7 mm for PMC, commercial samples harvested year-round exhibit extreme variation in thickness. While earlier studies suggested that specific diameter ranges might optimize THSG levels (Zhao et al., 2013), a comprehensive evaluation of how these diameter differences impact overall marker content in market samples remains lacking.

Second, the botanical authenticity of PMC is often challenged by infraspecific taxa and their complex nomenclatural history. *Polygonum multiflorum* var. *hypoleucum* (Ohwi) T.S. Liu, S.S. Ying & M.J. Lai (hereafter PMH), traditionally regarded as an endemic variety in Taiwan (Liu, Ying & Lai, 1976),has undergone several taxonomic reassignments. According to the latest circumscription in Plants of the World Online (Plants of the World Online, 2026), PMH and its alternative combinations in the genera *Fallopia* and *Reynoutria* are currently treated as taxonomic synonyms of PM.

Despite this taxonomic synonymy suggesting they are conspecific, PMH is still utilized as a distinct folk medicine in Taiwan under the name “Hong Ji Shi Teng” for indications such as influenza and nephritis. These uses differ from the clinical applications of PMC (Yang et al., 1999). Notably, while PMH is reported to contain various flavonoids and catechins, the presence of the characteristic constituent THSG remains unverified in this taxon (Chen et al., 2009). This discrepancy between botanical classification and chemical composition necessitates a rigorous re-evaluation of its medicinal status.

Morphologically, PM is a perennial twining herb characterized by large, black-brown, tuberous roots and slender, glabrous, striate stems bearing ovate, cordate-based leaves (Flora of China, 2025). In contrast, PMH lacks these enlarged tuberous roots but shows similar twining stems and glabrous, papillate-veined leaves, as described in the Flora of Taiwan (Liu, 1996). Because their vegetative stems and leaves are so similar, dried PM and PMH stems are difficult to distinguish visually, making additional chemical and molecular authentication necessary.

Similarly, *Pleuropterus angulatus* (S.Y. Liu) B.Q. Xu & G.H. Wen (syn. *Polygonum multiflorum* var. *angulatum*, hereafter PA), distinguished by its tetragonous branchlets and anomalous vascular strands, has recently been elevated to species rank (Liu, 1991; Wen et al., 2024; Yan et al., 2011).

Despite their morphological similarities to PMC, comprehensive chemical and pharmacological profiles for PA remain largely unexplored, creating a significant risk of inadvertent substitution in the market. This uncertainty about the chemical consistency among PMC, PMH stems (PMHC), and PA stems (PAC) highlights the urgent need for accurate authentication protocols.

The accuracy of such taxonomic and medicinal assessments relies heavily on robust molecular tools. The internal transcribed spacer 2 (ITS2) region has emerged as a standard barcode; however, it often exhibits significant intragenomic diversity across a wide range of organisms, from plants to vertebrates (Sánchez & Dorado, 2008). In many DNA sequencing studies, this variability has been overlooked, which may lead to inaccurate assessments of genetic diversity. Since Single Nucleotide Polymorphisms (SNPs) represent fundamental genomic variations linked to specific phenotypes, SNP analysis within the ITS2 region offers a superior approach for assessing genetic diversity and ensuring species differentiation, particularly among closely related medicinal taxa (Arif et al., 2010; Huq et al., 2016).

However, to date, no study has simultaneously addressed both the diameter-based quality standardization and the botanical authentication of PMC within a unified analytical framework, leaving a critical gap in pharmacognostic practice where neither chemical profiling alone nor morphological inspection can reliably distinguish authentic PMC from closely related adulterants such as PAC and PMHC.

In this study, we employed a dual-approach strategy integrating high-performance liquid chromatography (HPLC) for the quantification of three key marker compounds (THSG, emodin, and physcion) and ITS2 sequencing for molecular barcoding. By evaluating authenticated materials and commercial samples across various diameters, we aimed to: (1) clarify the impact of stem diameter on chemical potency, and (2) verify the true botanical origin of PMC available in Taiwan’s markets. These findings will enhance the accuracy of quality control frameworks and ensure the therapeutic reliability of PMC in modern clinical practice.

## Materials & methods

### Plant materials and crude drugs

A total of 42 batches of samples, comprising freshly harvested botanical specimens and dried crude drug materials, were analyzed in this study.

**Figure 1:**
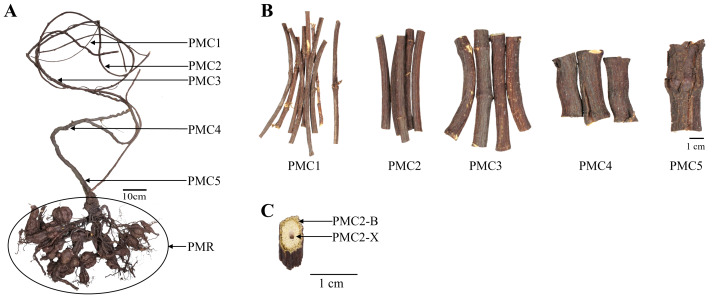
Morphological characterization and sampling strategy for *Pleuropterus multiflorus* (PM). (A) Crude drug form showing both stems (PMC) and the hallmark enlarged tuberous roots (PMR). (B) Representative PMC samples categorized into five groups based on stem diameter: 2.0–3.7 mm (PMC1), 4.6–6.6 mm (PMC2), 8.0–11.0 mm (PMC3), 12.1–15.0 mm (PMC4), and 17.0–25.1 mm (PMC5). (C) Cross-section of PMC2 showing the bark (PMC2-B) and xylem (PMC2-X). Scale bars: 10 cm (A), one cm (B, C).

**Figure 2:**
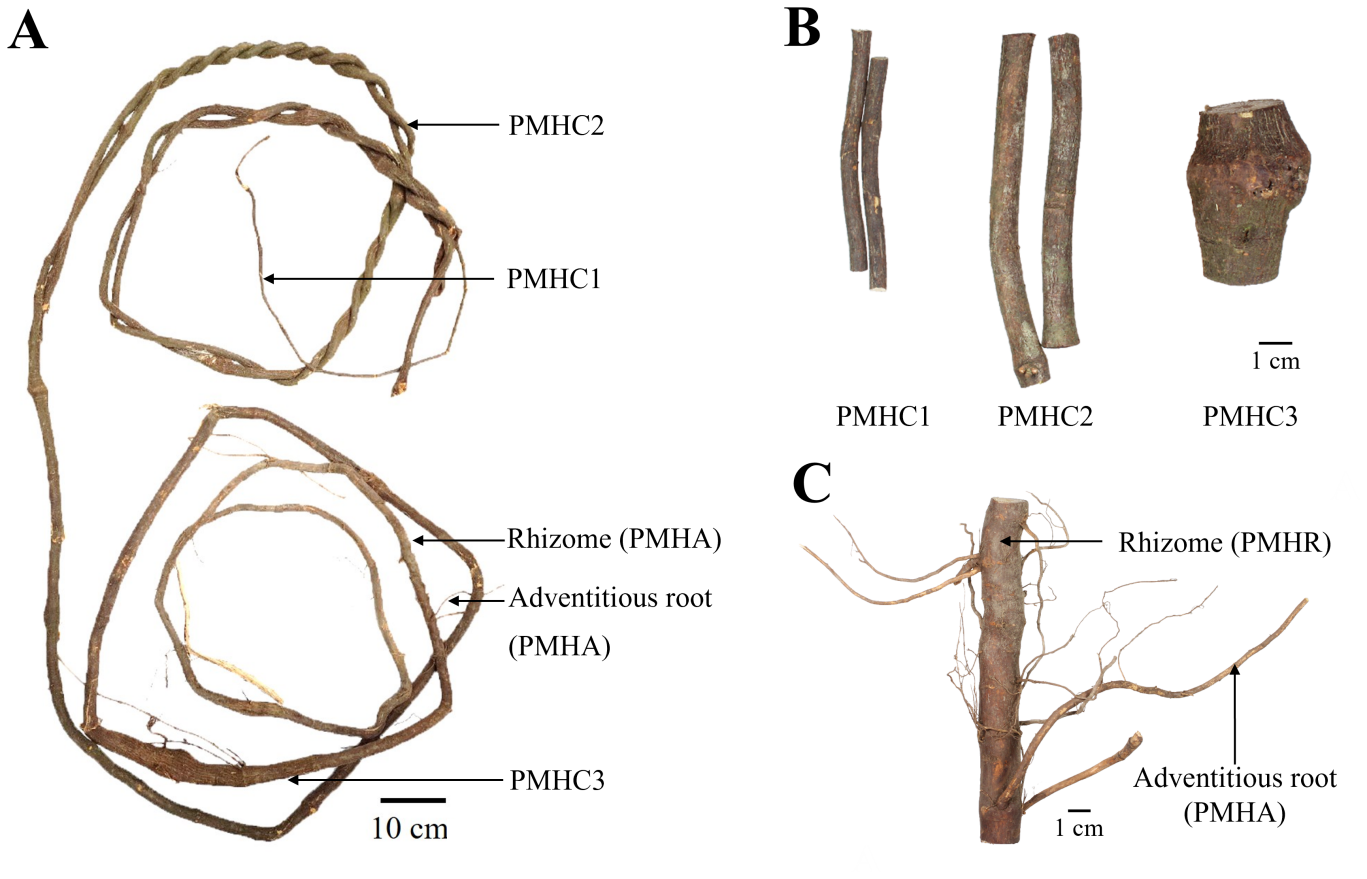
External morphology of *Polygonum multiflorum* var. *hypoleucum* (PMH). (A) Visual representation of the stems (PMHC1–3), rhizome (PMHR), and adventitious roots (PMHA). (B) Representative dried stem samples (PMHC1–3) categorized based on stem diameter: 2.4–2.7 mm (PMHC1), 6.5–10.0 mm (PMHC2), and 21.0–30.0 mm (PMHC3). (C) Detailed view of the underground structures, including the rhizome (PMHR) and adventitious roots (PMHA), highlighting the absence of enlarged tuberous roots as compared to *P. multiflorus*. Scale bars: 10 cm (A), one cm (B, C).

Fresh original plants of PM and PMH were harvested from Tainan (23°17′26.7″N, 120°20′23.3″E) and New Taipei City (24°52′05.7″N, 121°24′20.4″E), Taiwan, respectively. These fresh stems were subsequently air-dried and classified by diameter: PM stems were divided into five groups (PMC1–PMC5) (Figs. 1A and 1B), and PMH stems (PMHC) into three groups (PMHC1–PMHC3) (Fig. 2). To evaluate tissue-specific chemical distribution, the PMC2 group (4–7 mm) was further dissected into bark (PMC2-B) and xylem (PMC2-X) (Fig. 1C). For outgroup comparison, fresh leaves of *Reynoutria japonica* Houtt. (RJ) were collected from Taichung (24°09′22.7″N, 120°40′50.7″E).

**Figure 3:**
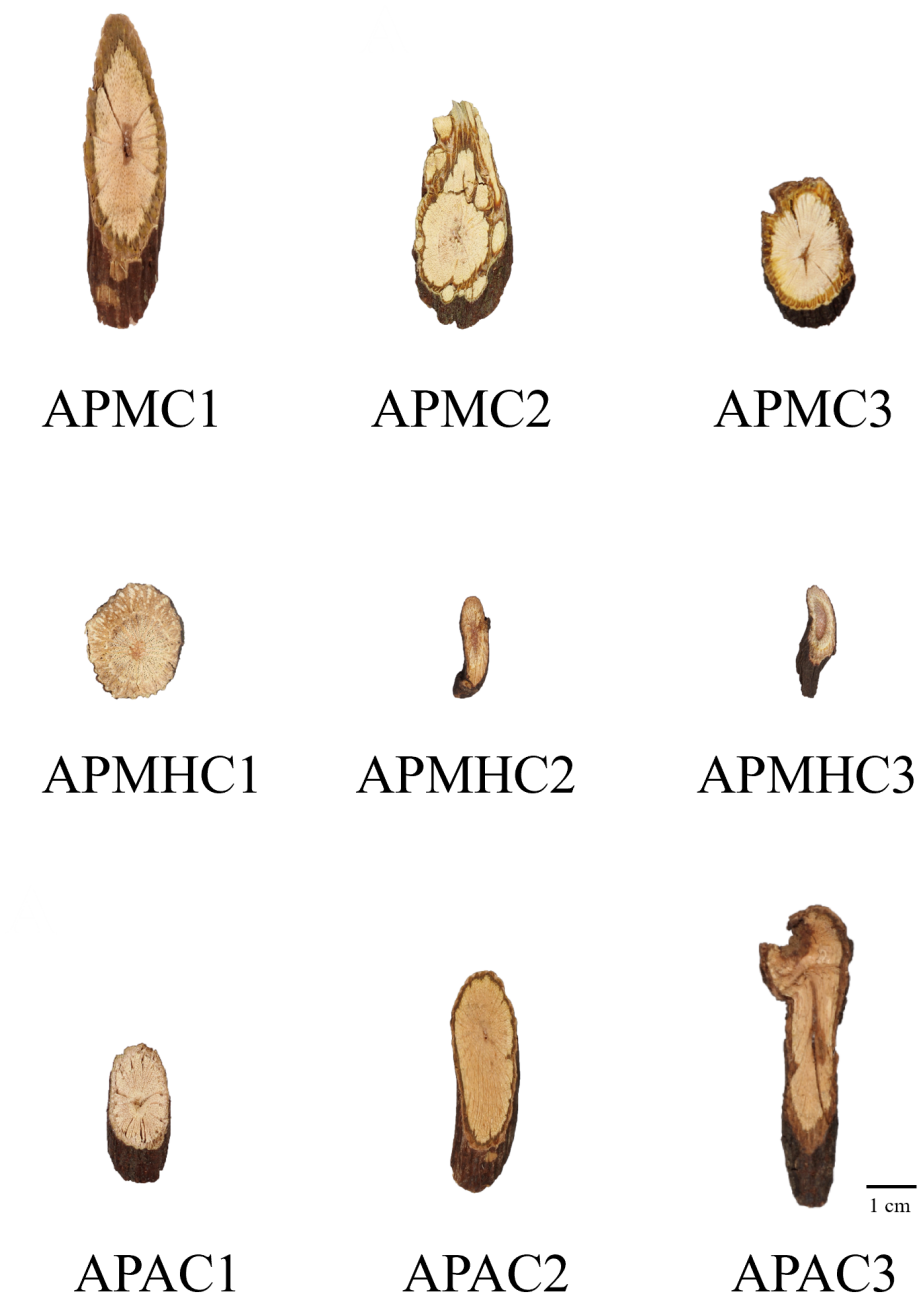
Comparative transverse sections of authenticated medicinal stems. Representative cross-sectional views of *Pleuropterus multiflorus* (APMC), *Polygonum multiflorum* var. *hypoleucum* (APMHC), and *Pleuropterus angulatus* (APAC) illustrating the distinct internal anatomical features that characterize each species.

Beyond the self-harvested specimens, dried authenticated crude drugs, including six batches of PMC (APMC1–6), three batches of PMHC (APMHC1–3), and ten batches of PA stems (APAC1–10) were acquired from various localities in Taiwan and China to account for geographical variation (Fig. 3). Additionally, eleven batches of commercial crude drug samples (CPMC1–11), labeled as Shou-Wu-Teng or Ye-Jiao-Teng, were randomly procured from traditional herbal stores in Taiwan.

All botanical origins were authenticated by Assistant Professor Kun-Chang Wu (China Medical University, Taichung, Taiwan) through morphological characterization and ITS2 sequencing. Voucher specimens (numbered CMURX23001–CMURX23034) are deposited at the School of Pharmacy, China Medical University, Taiwan. Scientific nomenclature follows Plants of the World Online (Plants of the World Online, 2026). Plant materials were collected from non-protected areas in Taiwan and China, and no specific permits were required for the collection activities according to local regulations. Commercial PMC samples were purchased from local herbal medicine markets.

### Reagents and chemicals

Reference standards of THSG (purity ≥98.0%) were purchased from the Shanghai R&D Center for Standardization of Traditional Chinese Medicines (Shanghai, China). Emodin (purity ≥98.7%) and physcion (purity ≥99.8%) were obtained from the National Institute for the Control of Pharmaceutical and Biological Products (NICPBP, Beijing, China). HPLC-grade methanol and acetonitrile were procured from Spectrum Chemical Mfg. Corp. (Shanghai, China), while analytical-grade formic acid was sourced from Honeywell Fluka (Germany). Ultra-pure water was prepared using a Synergy^®^ UV water purification system (Merck Ltd., Taipei, Taiwan). All other analytical-grade reagents were used without further purification.

### Quantitative chemical analysis

#### Preparation of standard solutions

Stock solutions of THSG (266 mg/L), emodin (101 mg/L), and physcion (122 mg/L) were individually prepared in methanol. A series of mixed working standard solutions was generated by serial dilution for calibration purposes. Calibration curves were constructed by plotting peak areas (*y*) against the respective concentrations (*x*, mg/L).

#### Extraction and sample preparation

Dried samples were pulverized into a fine powder (20 mesh). Approximately 0.2 g of the powdered material was accurately weighed and extracted with 20 mL of 75% (v/v) methanol in a 50 mL centrifuge tube. The mixture was subjected to ultrasonic extraction (Delta DC400H, 40 kHz) at 25 °C for 40 min. After centrifugation at 3,000 × *g* for 10 min, the supernatant was collected. This extraction process was repeated, and the combined supernatants were diluted to a final volume of 50 mL with 75% methanol. The extract was filtered through a 0.45 µm PTFE syringe filter (Hong Si Science, Ltd., Taichung, Taiwan) prior to an injection volume of 10 μL for HPLC analysis, following the established pharmacopeia protocols (Editorial Committee of the Hong Kong Chinese Materia Medica Standards, 2018).

#### HPLC-PDA analytical conditions

Chromatographic analysis was performed on a Shimadzu LC-40D XS System equipped with an SPD-M40 photodiode array (PDA) detector. Separation was achieved using a NUCLEODUR^®^ C18 Pyramid column (250 mm × 4.6 mm i.d., five µm; MACHEREY-NAGEL, USA). The mobile phase consisted of 0.5% (v/v) aqueous formic acid (A) and acetonitrile (B) with the following gradient elution: 0–18 min, 17% B; 18–30 min, 17–35% B; 30–40 min, 35% B; 40–50 min, 35–95% B; and 50–60 min, 95% B. The flow rate was maintained at one mL/min. UV–Vis spectra were monitored from 190 to 800 nm, and the column temperature was controlled at 35 °C. The chromatographic conditions, including the mobile phase composition, gradient programme, and detection wavelength, were adopted from the official HKCMMS assay for PMC (Editorial Committee of the Hong Kong Chinese Materia Medica Standards, 2018).

### DNA molecular analysis

#### Genomic DNA extraction

Homogenized tissues, pulverized in liquid nitrogen, were processed for total DNA extraction using the DNeasy Plant Mini Kit (QIAGEN, Hilden, Germany) according to the manufacturer’s instructions. DNA concentration and purity were quantified using a Qubit 4 fluorometer (Invitrogen). Isolated DNA was stored at −20 °C for downstream applications.

#### PCR amplification and ITS2 sequencing

The ITS2 region was amplified using primers ITS2-2F (5′-ATGCGATACTTGGTGTGAAT-3′) and ITS2-3R (5′-GACGCTTCTCCAGACTACAAT-3′). Each 20 μL PCR reaction contained 10 ng of template DNA, Phusion™ Plus Green PCR Master Mix (Thermo Fisher Scientific, USA), and 10 μM of each primer. Thermal cycling (Bio-Rad MyCycler) involved: initial denaturation at 98 °C for 1 min; 35 cycles of 98 °C (10 s), 55 °C (10 s), and 72 °C (1 min); and a final extension at 72 °C for 5 min. PCR products were analyzed *via* 2.2% agarose gel electrophoresis and subsequently sequenced.

Sequencing was performed in a single direction using the forward primer ITS2-2F. As the ITS2 region (∼193 bp) is centrally located within the amplicon, single-direction sequencing provides complete and reliable coverage of the target region. The raw sequence files (.ab1) were processed through the ITS2 Database (http://its2.bioapps.biozentrum.uni-wuerzburg.de) (Keller et al., 2009) for Hidden Markov Model (HMM)-based annotation and excision of the flanking 5.8S and 28S rRNA regions, and the resulting sequences were verified by manual inspection of the electropherograms prior to downstream analysis.

#### SNP identification and genotyping

To evaluate intragenomic and intraspecific variability, ITS2 sequences were screened *via* BLAST (Basic Local Alignment Search Tool) and processed through HMM analysis to excise conserved 5.8S and 28S rRNA regions (Keller et al., 2009). SNPs were identified using NovoSNP 3.0.1 (Weckx et al., 2005), with sequencing files (.ab1) aligned against the *P. multiflorus* reference (GenBank: KM279320). A quality-score cutoff of ≥15 was applied for SNP detection, based on the benchmark analysis of Weckx et al. (2005), which demonstrated that this threshold retained 84.3% of true variants while substantially reducing false positives, representing a practical balance between sensitivity and specificity in resequencing data. To further ensure result reliability, potential artifacts were excluded by manual inspection of the raw chromatographic data (electropherograms) for each variable site and its adjacent bases. Validated polymorphisms were categorized into SNP genotypes for species differentiation.

#### Phylogenetic reconstruction

ITS2 sequences from authenticated samples were aligned using Clustal W. Phylogenetic relationships were reconstructed *via* the Neighbor-Joining (NJ) method in MEGA 11 with 1,000 bootstrap replicates to assess nodal support (Saitou & Nei, 1987; Tamura, Stecher & Kumar, 2021). Genetic divergence was quantified through mean intra- and interspecific *p*-distances (Felsenstein, 1985).

## Results

### Impact of stem diameter and tissue localization on constituent accumulation in PMC

We conducted HPLC analyses on five dry sample groups (PMC1–PMC5) with diameters spanning from 2.0 to 25.1 mm harvested from a single PM plant (Fig. 1). The external standard calibration curves displayed high linearity for THSG (*y* = 15674*x* − 23054, *R*^2^ = 0.9997), emodin (*y* = 37618*x* − 595.15, *R*^2^ = 1.0000), and physcion (*y* = 18641*x* − 14571, *R*^2^ = 0.9983). As summarized in Table 1, the results indicated that specimens with diameters exceeding seven mm (PMC3, PMC4, and PMC5) possessed higher THSG levels compared to those with diameters below seven mm (PMC1 and PMC2). While emodin and physcion levels were more prominent in PMC1, PMC2, and PMC3 than in PMC4 and PMC5, all samples across the 2.0–25.1 mm range successfully met the regulatory standards of the THP, CP, and HKCMMS. Furthermore, tissue-specific analysis of PMC2 indicated that THSG, emodin, and physcion are primarily concentrated in the bark (PMC2-B) rather than the xylem (PMC2-X).

**Table 1:** Quantitative analysis of 2,3,5,4′-tetrahydroxystilbene-2-O-*β*-D-glucoside (THSG), emodin, and physcion in *Pleuropterus multiflorus* (PM) stems across varying diameters and anatomical tissues.

**Sample** **number**	**Diameter** **(mm)**	**Location**	Content (%, dry weight)*			**Moisture** **content (%)**	**Accession** **number**
			**THSG**	**Emodin**	**Physcion**		
PMC1	2.0–3.7	Tainan	1.85	0.071	0.132	9.61	PQ481778
PMC2	4.6–6.6			3.04	0.122	0.131		9.62	
PMC3	8.0–11.0			4.43	0.153	0.148		8.99	
PMC4	12.1–15.0			4.22	0.017	0.043		8.29	
PMC5	17.0–25.1			4.16	0.012	0.041		10.62	
PMC2-B	_			4.45	0.277	0.245		9.62	
PMC2-X	_		0.89	0.012	0.031	9.62	

**Notes.**

PMC the stem of *Pleuropterus multiflorus* (PM) PMC2-B the bark of PMC2 PMC2-X the xylem of PMC2 * The contents were calculated on a dry weight basis _ not measured

### Chemotaxonomic analysis of PMH and its distinction from PMC

Although current botanical authorities treat PMH as a synonym of PM (Plants of the World Online, 2026), our comprehensive profiling revealed a profound lack of pharmacognostic equivalence between these two taxa. Quantitative chemical analysis across various tissues of the original PMH plant, including the stems (PMHC1–3), rhizomes (PMHR), and adventitious roots (PMHA), demonstrated that THSG was consistently undetectable regardless of the organ type or stem diameter (Fig. 2, Table 2). While emodin and physcion were identified in specific tissues such as the larger stems (PMHC3) and adventitious roots (PMHA), the absolute absence of the characteristic compound THSG serves as a definitive chemical marker distinguishing PMH from the official PMC.

**Table 2:** Concentrations of THSG, emodin, and physcion in the stems, rhizomes, and adventitious roots of *Polygonum multiflorum* var. *hypoleucum* (PMH) categorized by tissue type and diameter.

**Sample** **number**	**Diameter** **(mm)**	**Location**	Content (%, dry weight)*			**Moisture** **content (%)**	**Accession** **number**
			**THSG**	**Emodin**	**Physcion**		
PMHC1	2.4–2.7	New Taipei	ND	ND	ND	5.97	PQ481779
PMHC2	6.5–10.0			ND	ND	ND		6.12	
PMHC3	21.0–30.0			ND	0.002	0.031		6.85	
PMHR	12.7–18.6			ND	0.003	0.040		6.84	
PMHA	0.3–2.8		ND	0.028	0.110	7.31	

**Notes.**

PMHC the stem of PMH PMHR the rhizome of PMH PMHA the adventitious root of PMH * The contents were calculated on a dry weight basis ND not detected

Furthermore, the morphological observations recorded in this study provide crucial physical evidence to supplement the chemical findings. As illustrated in Fig. 2A, the PMH plant lacks the characteristic enlarged tuberous roots, known as Pleuropteri Multiflori Radix (PMR), which are the hallmark morphological feature of PM.

### Integrated chemical and genetic authentication framework

Due to the morphological similarities between PMC, PMHC, and PAC, an integrated approach combining HPLC fingerprinting and ITS2 barcoding was developed to ensure precise quality control and prevent unintentional substitution.

#### Chemical phenotyping of authenticated samples

**Figure 4:**
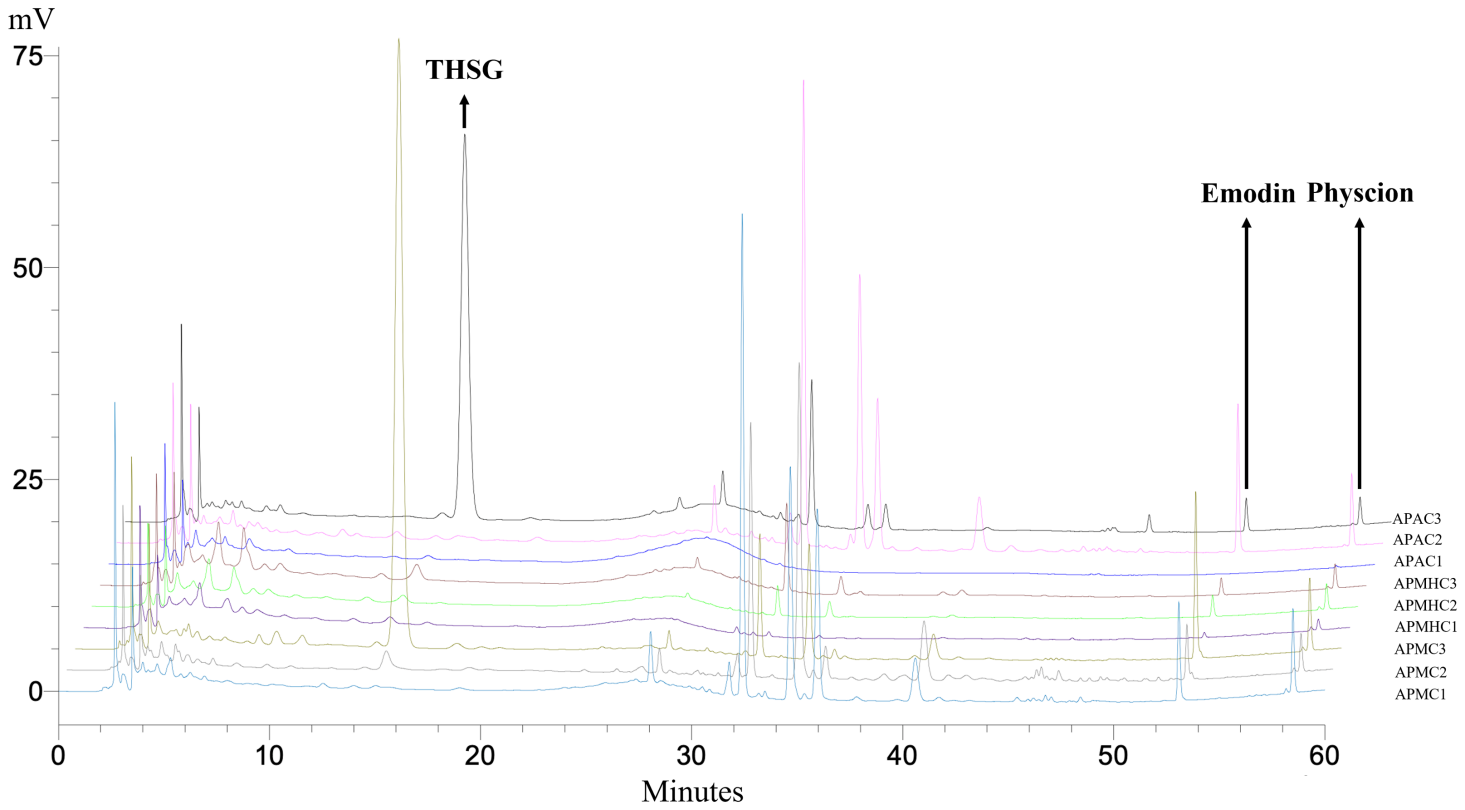
HPLC chromatographic profiles of nine authenticated stem samples. Representative chromatograms of *Pleuropterus multiflorus* (APMC1–3), *Polygonum multiflorum* var. *hypoleucum* (APMHC1–3), and *Pleuropterus angulatus* (APAC1–3) showing the varying concentrations of THSG, emodin, and physcion across the different species and chemical phenotypes. Black arrows indicate the characteristic peaks of THSG, emodin, and physcion at their respective retention times.

The quantitative analysis of nine authenticated batches (Fig. 3), including PMC (APMC1–3), PMH (APMHC1–3), and PAC (APAC1–3), revealed significant chemotypic diversity among these taxa, as summarized in Table 3. The corresponding HPLC chromatographic patterns presented in Fig. 4 further illustrate the distinct chemical profiles and varying analyte distributions across the samples.

**Table 3:** Comparative chemical profiling of THSG, emodin, and physcion in authenticated samples of APMC, APMHC, and APAC highlighting distinct chemical phenotypes.

**Sample** **number**	**Diameter** **(mm)**	**Chemical phenotypes**	**Species**	Content (%, dry weight)*			**Accession** **number**
				**THSG**	**Emodin**	**Physcion**	
APMC1	1.5–1.7	PMC type I	PM	ND	0.058	0.119	PQ452593
APMC2	1.9–2.0	PMC type II			0.13	0.035	0.067	PQ452594
APMC3	1.8–1.9			2.85	0.109	0.110	PQ452595
APMHC1	1.8–2.1	PMHC type	PMH	ND	0.004	0.034	PQ452599
APMHC2	0.4–0.5					ND	0.014	0.056	PQ452600
APMHC3	0.5–0.7			ND	0.003	0.040	PQ452601
APAC1	1.1–1.2	PAC type I	PA	ND	ND	ND	PQ452602
APAC2	1.1–1.3				ND	0.077	0.102	PQ452603
APAC3	1.1–1.9	PAC type II		2.02	0.021	0.046	PQ452604

**Notes.**

APMC the stem of the authenticated PM sample APMHC the stem of the authenticated PMH sample APAC the stem of the authenticated *Pleuropterus angulatus* (PA) sample * The contents were calculated on a dry weight basis ND not detected

Within the APMC group, a notable intraspecific variation in THSG content was observed, leading to the identification of two chemical phenotypes. PMC Type I (represented by APMC1) was characterized by the complete absence of THSG, despite containing emodin (0.058%) and physcion (0.119%). In contrast, PMC Type II (APMC2 and APMC3) exhibited detectable THSG levels, with APMC3 reaching a high concentration of 2.85%. Notably, only the APMC3 sample fulfilled the stringent potency requirements established by the CP, THP, and HKCMMS, highlighting that botanical authenticity does not invariably guarantee regulatory compliance.

Regarding the APMHC samples, the chemical profiles were consistently distinguished by the total absence of THSG across all three batches (APMHC1–3). While these samples yielded trace to low amounts of emodin (0.003%–0.014%) and physcion (0.034%–0.056%), the lack of the primary bioactive marker THSG underscores a critical pharmacognostical divergence from the official PMC.

The APAC samples exhibited the most complex chemotypic profiles, which were further categorized into two phenotypes. PAC Type I included APAC1 (in which all three analytes were undetectable) and APAC2 (containing only anthraquinones). More significantly, PAC Type II (represented by APAC3) was found to contain a substantial THSG level of 2.02%, alongside emodin (0.021%) and physcion (0.046%).

#### ITS2 sequence variation and phylogenetic resolution

**Figure 5:**
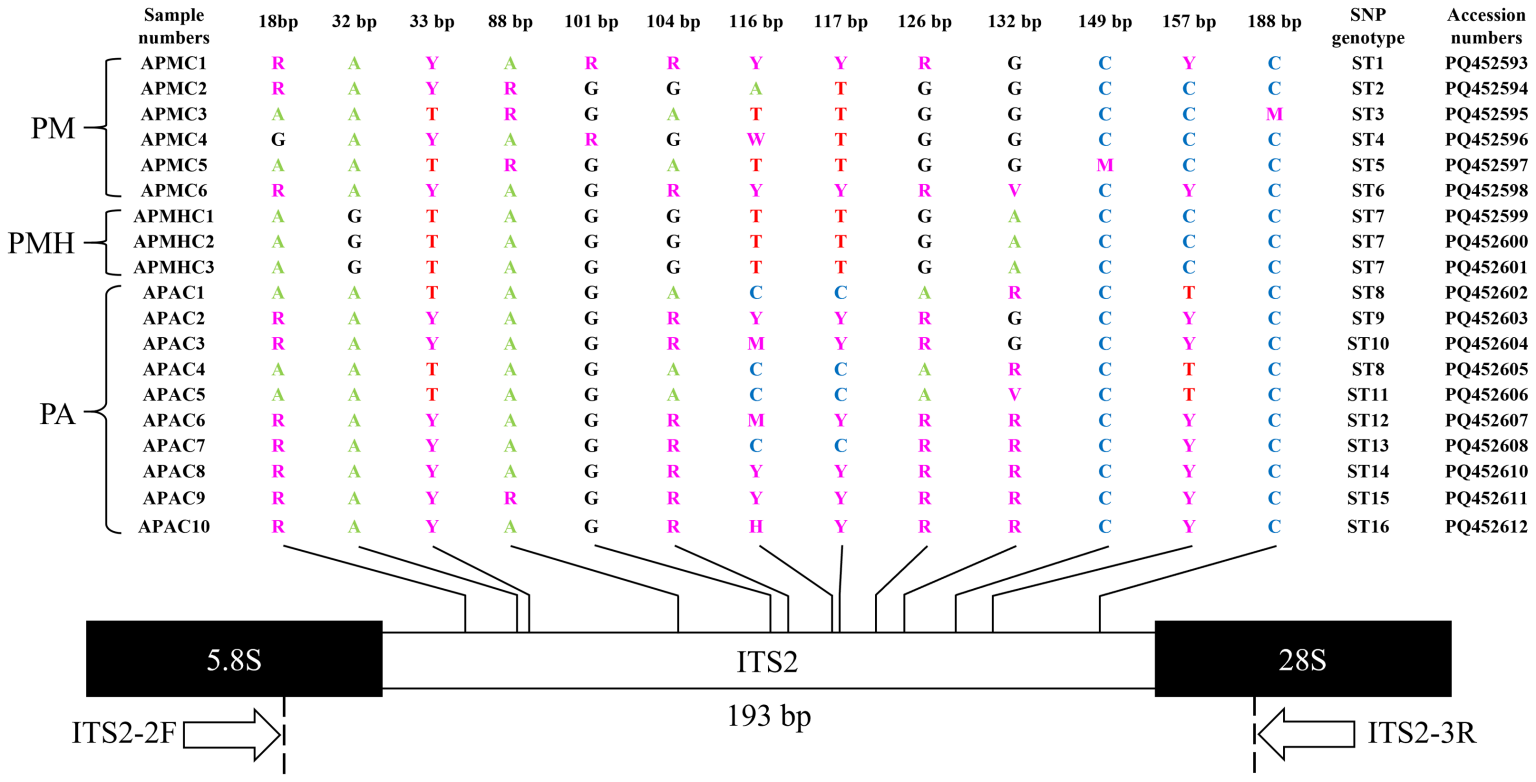
Distribution and identification of 13 SNPs within the ITS2 region. Numerical labels above the sequences represent nucleotide positions of the SNPs. Heterozygous sites are defined according to IUPAC nomenclature, where W = A/T, M = A/C, R = A/G, Y = C/T, V = A/C/G, H = A/C/T.

To resolve the phylogenetic relationships among the analyzed taxa, we expanded the sampling of APMC, APMHC, and APAC to capture a broader range of genetic diversity. Sequencing of the ITS2 region yielded a consistent length of 193 bp characterized by a remarkably high density of SNPs. A detailed analysis identified 13 SNP sites spanning from 18 bp to 188 bp, which collectively defined 16 distinct SNP genotypes among the authenticated samples (Fig. 5). High levels of heterozygosity were observed at nine specific positions, such as 18, 33, 88, 104, 116, 117, 126, 132, and 157 bp, reflecting complex intragenomic variation within these species.

**Figure 6:**
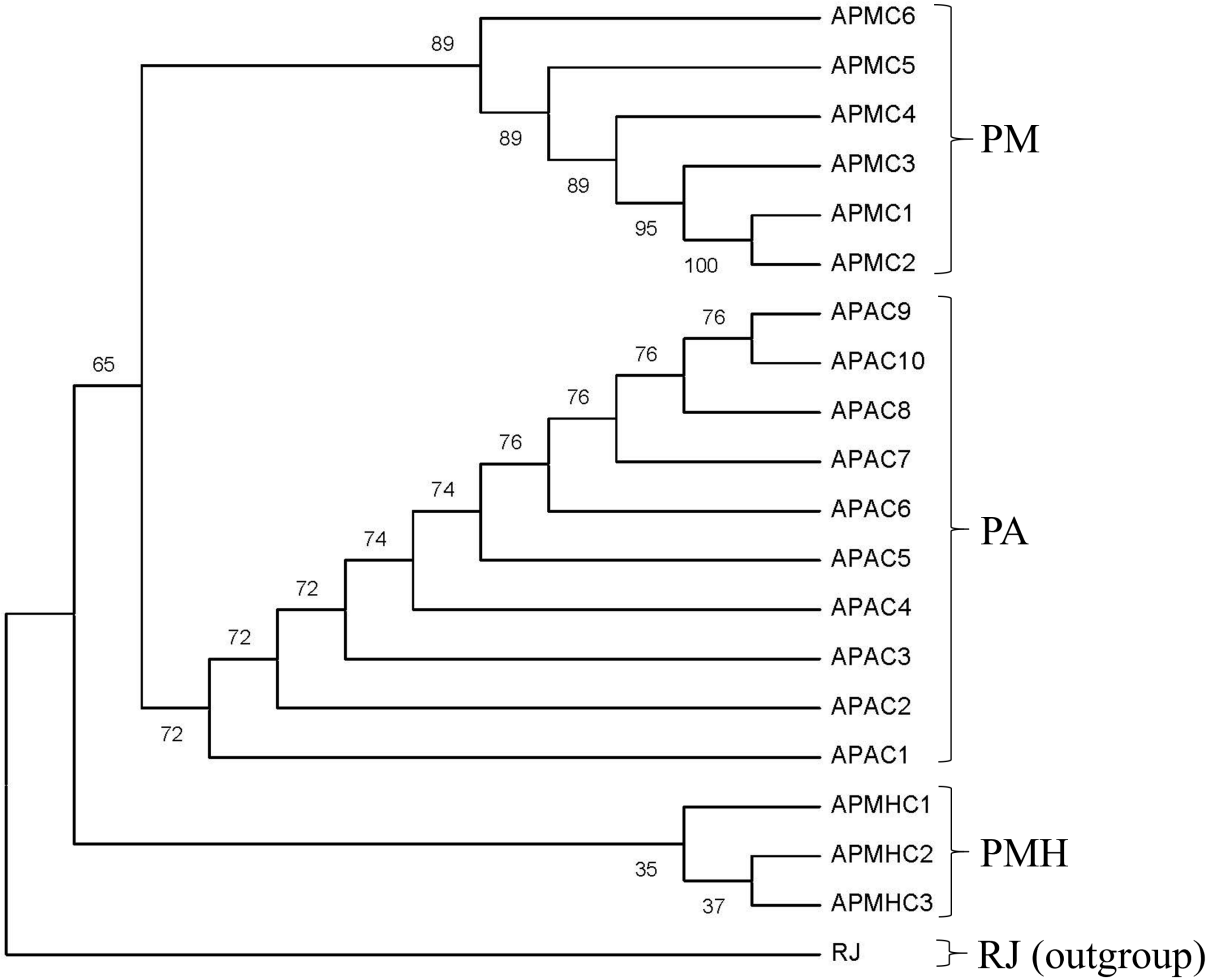
Neighbor-Joining (NJ) phylogenetic tree based on ITS2 sequences. Genetic relationships among PM, PMH, and PA samples are resolved with 1,000 bootstrap replicates. Reynoutria japonica (RJ) is utilized as the outgroup to provide evolutionary context.

The NJ phylogenetic tree (Fig. 6), supported by 1,000 bootstrap replicates, partitioned the samples into three monophyletic clades corresponding to PM, PMH, and PA. The high bootstrap values at the main nodes, together with the inclusion of RJ as an outgroup, support the high discriminatory power of the ITS2 region for these taxa. This genetic resolution effectively bridges the analytical gap where chemical profiling alone is insufficient, providing a rigorous molecular framework for the differentiation of PMC from its closely related congeners.

### Market survey and authentication of commercial PMC samples

A comprehensive market survey of eleven commercial PMC (CPMC) samples across Taiwan revealed a significant discrepancy between labeled identity and botanical reality. Integrated ITS2 sequencing identified that a staggering 82% (nine out of 11) of the market samples were actually derived from PA, whereas only two samples were confirmed as genuine PM (Table 4).

**Table 4:** Integrated ITS2 sequencing results and quantitative analysis of marker compounds in eleven commercial batches of Polygoni Multiflori Caulis (CPMC) from Taiwanese herbal stores.

**Sample** **number**	**Labeled** **name**	**Location**	**ITS2 sequencing** **result**	**Chemical** **phenotype**	Content (%, dry weight)*			**Accession** **number**
					**THSG**	**Emodin**	**Physcion**	
CPMC1	PMC	Changhua	PA	PAC type I	ND	ND	ND	PQ452613
CPMC2	PMC	Taichung	PM	PMC type II	3.25	0.185	0.249	PQ452614
CPMC3	PMC	Taichung	PA	PAC type I	ND	ND	ND	PQ452615
CPMC4	PMC	Jiayi	PA	PAC type I	ND	ND	ND	PQ452616
CPMC5	PMC	Kaohsiung	PA	PAC type II	2.01	0.09	0.256	PQ452617
CPMC6	PMC	Taichung	PA	PAC type II	ND	ND	ND	PQ452618
CPMC7	PMC	Taichung	PA	PAC type I	ND	ND	ND	PQ452619
CPMC8	PMC	Jiayi	PA	PAC type II	2.88	0.007	0.035	PQ452620
CPMC9	PMC	Jiayi	PA	PAC type II	ND	ND	ND	PQ452621
CPMC10	PMC	Kaohsiung	PM	PMC type II	3.16	0.061	0.107	PQ452622
CPMC11	PMC	Kaohsiung	PA	PAC type II	ND	ND	ND	PQ452623

**Notes.**

CPMC commercial PMC sample, all claiming to be Shou-Wu-Teng or Ye-Jiao-Teng by providers * The contents were calculated on a dry weight basis ND not detected

The chemical potency of these market samples varied drastically, with THSG levels ranging from undetected (ND) to 3.25%. Notably, while the two genuine PMC samples, CPMC2 and CPMC10, exhibited superior THSG concentrations exceeding 3.1%, 72% of the PA-adulterated samples failed to yield any detectable levels of the three key analytes. Although a small subset of PA samples, specifically CPMC5 and CPMC8 (classified as PAC Type II), managed to meet THP standards, their overall chemical profiles remained inconsistent. The high prevalence of PA as a substitute, which often lacks essential secondary metabolites, not only compromises therapeutic efficacy but also highlights a critical vulnerability in the current TCM supply chain.

## Discussion

In the history of plant taxonomy, PMH and PA were frequently regarded as infraspecific variants of PM. While some recent botanical circumscriptions treat PMH and PA as taxonomic synonyms of PM based on morphological similarities (Editorial Committee of Flora Reipublicae Popularis Sinicae, 1998), the latest authorities, such as POWO, have reinstated PA as an independent species (Plants of the World Online, 2026). Such changes in scientific nomenclature often create ambiguity in the herbal trade, as botanists primarily focus on morphological or molecular affinity rather than the concentration of bioactive markers, such as THSG, emodin, and physcion. Consequently, this study employs an integrated chemical and genetic framework to evaluate whether these closely related taxa can be considered legitimate medicinal substitutes for PMC.

A critical finding of this study involves revising the quality criteria regarding stem diameter. Previous literature and several pharmacopoeias have suggested that a diameter range of 4–7 mm is essential for ensuring optimal quality in PMC (Chinese Pharmacopoeia Commission, 2025; Editorial Committee of the Hong Kong Chinese Materia Medica Standards, 2018; Editorial Committee of the Taiwan Herbal Pharmacopoeia, 2021; Zhao et al., 2013). However, our systematic analysis of stems from the same original PM plant (Fig. 1 and Table 1) contradicts this convention. We observed that stems exceeding seven mm in diameter tended to contain higher concentrations of the three target analytes than those within the recommended range. This discovery may challenge the necessity of the 4–7 mm diameter threshold and suggests that PMC quality could be assessed based on the ontogenetic development of the specific plant rather than arbitrary size categories. Such a potential expansion of the acceptable diameter range might contribute to the more sustainable utilization of medicinal resources and the optimization of herbal production. However, we note that a broader diameter range may increase variability in active-compound levels per unit dose, and any further extension beyond the currently adopted 4–7 mm range should be supported by additional clinical and pharmacokinetic data before being implemented in practice.

The observed chemical variability within genuine PM samples may be attributed to the differential expression of biosynthetic genes. Specifically, the enzymes *FmUGT1* and *FmUGT2* have been identified as key catalysts in the glycosylation process that forms THSG. Given that the downregulation of these genes *via* RNA interference leads to a significant reduction in THSG content (Cai et al., 2022), it is plausible to speculate that the noncompliance of APMC1 and APMC2 samples with pharmacopoeial standards may be associated with the low expression levels of these specific transferases. This genetic regulation underscores the importance of verifying the chemical phenotype even when the botanical origin is authentic. Future transcriptomic studies would be needed to confirm this hypothesis.

It is also worth acknowledging that environmental factors may have contributed to the observed phytochemical variability. Meteorological variables, particularly temperature and altitude, have been reported to influence the accumulation of THSG and emodin in PM roots, with annual average temperature showing a negative correlation with THSG content and a positive correlation with emodin, while higher altitudes were associated with greater THSG accumulation (Son et al., 2025). These findings suggest that the chemical variability observed in our samples may reflect not only genetic divergence but also the influence of growing conditions.

Regarding the medicinal feasibility of PMH, our study provides a multifaceted rejection of its use as a substitute for PM. As illustrated in Fig. 2, PMH fails to develop the characteristic enlarged tuberous roots that are the hallmark of PM, which means it cannot serve as a source for the significant TCM known as PMR. This morphological deficiency is compounded by our chemical data in Tables 2 and 3, which confirm the total absence of THSG across all PMH tissues. The combination of these structural and phytochemical discrepancies provides strong evidence that, within the scope of the samples examined in this study, PMH is not pharmacognostically equivalent to PM. Accordingly, our results do not support the inclusion of PMH within the official medicinal circumscription of PMC and PMR.

The chemical diversity observed in PAC samples further complicates the quality control of PMC. Notably, although some PAC individuals (Type II) produced THSG at levels sufficient to pass pharmacopoeial screening (*e.g.*, CP and THP), their botanical identity remained distinct from those of genuine PMC, presenting a particularly deceptive challenge for routine chemical-based quality control. This finding underscores that chemical profiling alone is insufficient for unambiguous species authentication, and highlights the indispensable role of ITS2 SNP barcoding as a complementary genetic tool in the proposed dual-verification framework. More broadly, while PMH may still be debated as an intraspecific variant, our data demonstrate that both PMH and PA lack the chemical consistency required for therapeutic interchangeability (Figs. 3 and 4, and Table 3). The high density of 13 SNPs and 16 distinct SNP genotypes identified in our ITS2 analysis (Figs. 5 and 6) provides a powerful molecular tool to resolve these lineages. This genetic framework advances our understanding of the evolutionary dynamics between these species and establishes a foundation for future research into the functional genomics of THSG biosynthesis.

For PMC, our results further show that neither dataset alone can guarantee that a given batch is pharmacognostically acceptable. PMC Type I (*e.g.*, APMC1) is genetically authentic PM in the ITS2 SNP tree but fails to satisfy pharmacopoeial THSG requirements, whereas PAC Type II samples display non-PM ITS2 genotypes yet can occasionally meet pharmacopoeial THSG levels, and PMH consistently lacks THSG despite being closely related to PM. In practical terms, HPLC quantification ensures compliance with marker content and can effectively flag PMH or low-quality PA chemotypes with absent or substandard THSG, but it cannot exclude PA individuals that incidentally reach pharmacopoeial thresholds. Conversely, ITS2 barcoding secures species identity (PM *vs* PMH *vs* PA) but does not by itself assure that a given PM batch fulfills pharmacopeial marker requirements. By requiring both PM-specific ITS2 SNP genotypes and pharmacopoeial HPLC marker levels, the integrated framework therefore provides a stricter, mutually reinforcing authentication criterion than either method used in isolation.

The challenges of chemical variability driven by genetic divergence are well-documented in other high-value medicinal plants. For instance, the distinction between drug-type and fiber-type *Cannabis sativa* (Kojoma et al., 2006) as well as the variability among *Curcuma* species (Chen et al., 2023) demonstrates how genetic backgrounds dictate the synthesis of bioactive secondary metabolites (Ayer et al., 2018; Sawler et al., 2015). Our integrated approach utilizing HPLC and ITS2 sequencing has revealed for the first time that a staggering 82% of commercial PMC in the market is actually PA, predominantly the low-quality PAC Type I (Table 4). This high rate of adulteration not only compromises the clinical efficacy of TCM for insomnia but also calls into question the validity of previous pharmacological studies that may have utilized misidentified materials.

These findings carry significant practical implications for both regulatory authorities and the herbal medicine industry. For regulatory bodies, the evidence supporting an expanded acceptable stem diameter range of 2–25 mm may provide a scientific basis to inform future revisions of pharmacopoeial specifications, potentially optimizing the sustainable utilization of medicinal resources without compromising product quality. Furthermore, the high prevalence of PA substitution (82%) identified in this market survey highlights the limitations of relying solely on morphological inspection and suggests the potential value of incorporating integrated chemical-genetic authentication, such as the HPLC–ITS2 SNP barcoding approach proposed here, into official quality control frameworks. For industry stakeholders and herbal suppliers, these findings provide important evidence regarding the widespread adulteration of commercial PMC and emphasize the importance of adopting rigorous source verification protocols to safeguard therapeutic efficacy and consumer safety.The empirical results reported in this study should be viewed in light of some constraints. Our analyses were based on a finite number of authenticated plants and commercial batches. Nevertheless, the convergent chemical and ITS2 SNP evidence provides a consistent picture of chemotypic differentiation and species discrimination, and future studies extending this integrated framework to larger, geographically broader sample sets and incorporating additional markers will be important to further generalize and refine these findings.

## Conclusions

To the best of our knowledge, this work represents the first comprehensive investigation into the medicinal quality and authentication of PMC, PMHC, and PAC through the integration of HPLC-PDA quantification and ITS2 SNP barcoding. Our results indicate that PMC stems with diameters from 2–25 mm can satisfy existing pharmacopoeial marker requirements, suggesting a potentially broader usable range than the traditional 4–7 mm; however, this proposed interval should be validated across additional individuals and cultivation regions before any formal pharmacopoeial revision is considered. Furthermore, the significant morphological and chemical discrepancies found in PMH and PA, particularly the absence of tuberous roots and the inconsistent presence of THSG, indicate that these taxa are unlikely to serve as appropriate alternative botanical sources for PMC based on the current evidence. The dual-verification strategy proposed in this study effectively ensures the authenticity and therapeutic potency of PMC in both clinical and commercial settings.

##  Supplemental Information

10.7717/peerj.21561/supp-1 Supplemental Information 1 Raw dataset for the chemical and genetic authentication analyses of Polygoni Multiflori Caulis Each data point represents the raw measurement obtained from individual Polygoni Multiflori Caulis samples. The dataset includes unprocessed values used for chemical profiling and genetic authentication. All entries correspond directly to the analyses reported in the manuscript, allowing full reproducibility of the results.

## List of abbreviations

APAC: Authenticated stems of PA
APMC: Authenticated PMC
APMHC: Authenticated stems of PMH
BLAST: Basic Local Alignment Search Tool
CP: Chinese Pharmacopoeia
CPMC: Commercial PMC
HKCMMS: Hong Kong Chinese Materia Medica Standards
HMM: Hidden Markov Model
HPLC: High-performance liquid chromatography
ITS2: Internal transcribed spacer 2
ND: Not detected
NJ: Neighbor-Joining
PA: *Pleuropterus angulatus*
PAC: Stems of *Pleuropterus angulatus*
PCR: Polymerase chain reaction
PDA: Photodiode array
PM: *Pleuropterus multiflorus*
PMC: Polygoni Multiflori Caulis
PMH: *Polygonum multiflorum* var. *hypoleucum*
PMHC: Stems of *Polygonum multiflorum* var. *hypoleucum*
PMR: Pleuropteri Multiflori Radix
POWO: Plants of the World Online
RJ: *Reynoutria japonica*
SNPs: Single nucleotide polymorphisms
TCM: Traditional Chinese medicine
THP: Taiwan Herbal Pharmacopoeia
THSG: 2,3,5,4′-tetrahydroxystilbene-2-*O*-*β*-D-glucoside

## Additional Information and Declarations

### Data Availability

The following information was supplied regarding data availability: The data is available in the Supplemental Files. The raw sequence data are available at NCBI BioProject: PQ481778, PQ481779, PQ452593 to PQ452608, PQ452610 to PQ452612, PQ452613 to PQ452623.
