# Supplementary material for: Integrated chemical and genetic authentication of Polygoni Multiflori Caulis: Refuting diameter restrictions and exposing market adulteration
Source: PeerJ. 2026 Aug 3;14:e21561. doi: 10.7717/peerj.21561 (PMC13446225; doi:10.7717/peerj.21561)

Peerj Supplimentary data

Figure 1. External morphology and cross-section of *Pleuropterus multiflorus* (PM)
(A) The crude form of PM consists of both stems and tuberous roots (PMR). (B) PMC samples were collected and divided into 5 groups on the basis of their stem diameters: 2.0–3.7 mm (PMC1), 4.6–6.6 mm (PMC2), 8.0–11.0 mm (PMC3), 12.1–15.0 mm (PMC4), and 17.0–25.1 mm (PMC5). (C) The cross section of PMC2 includes the bark (PMC2-B) and the xylem (PMC2-X).

(A)

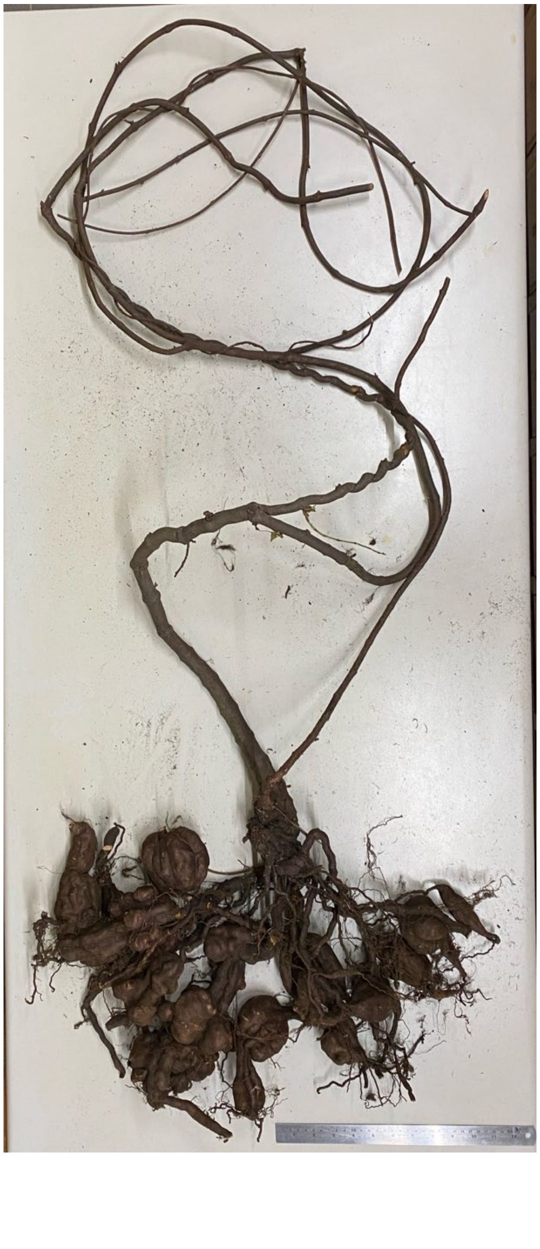

(B)

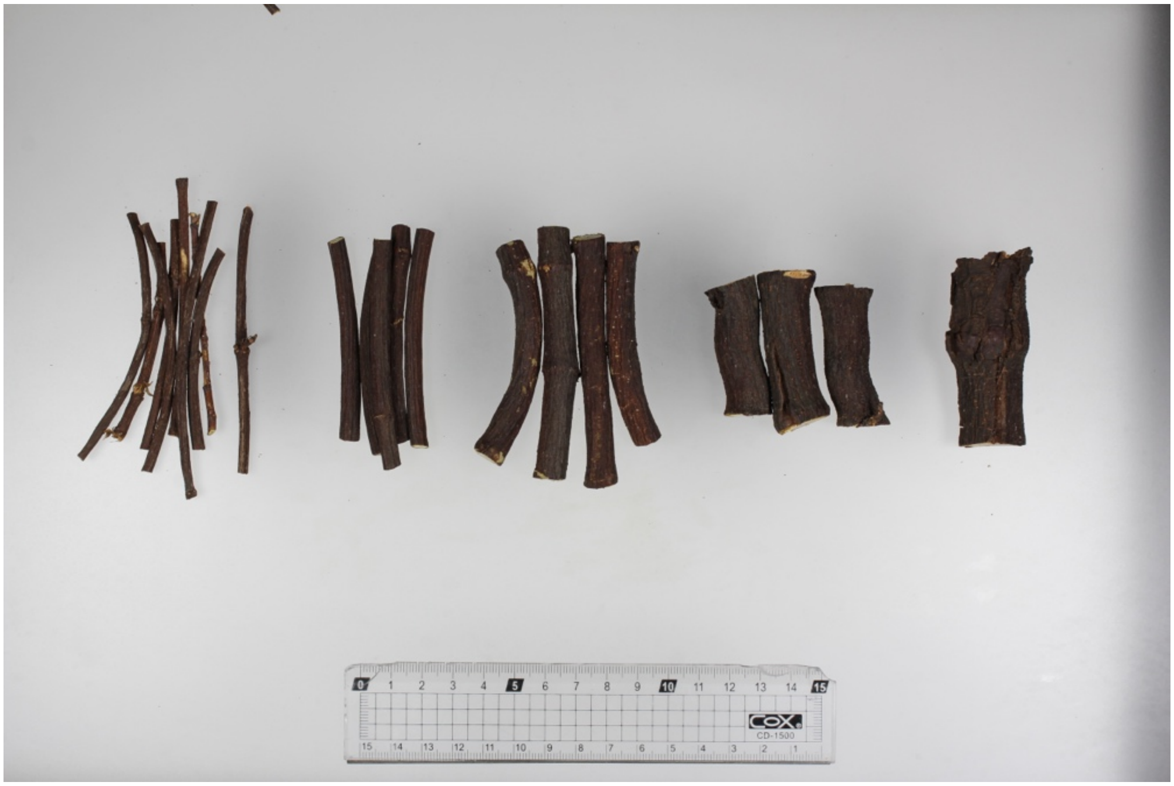

(C)

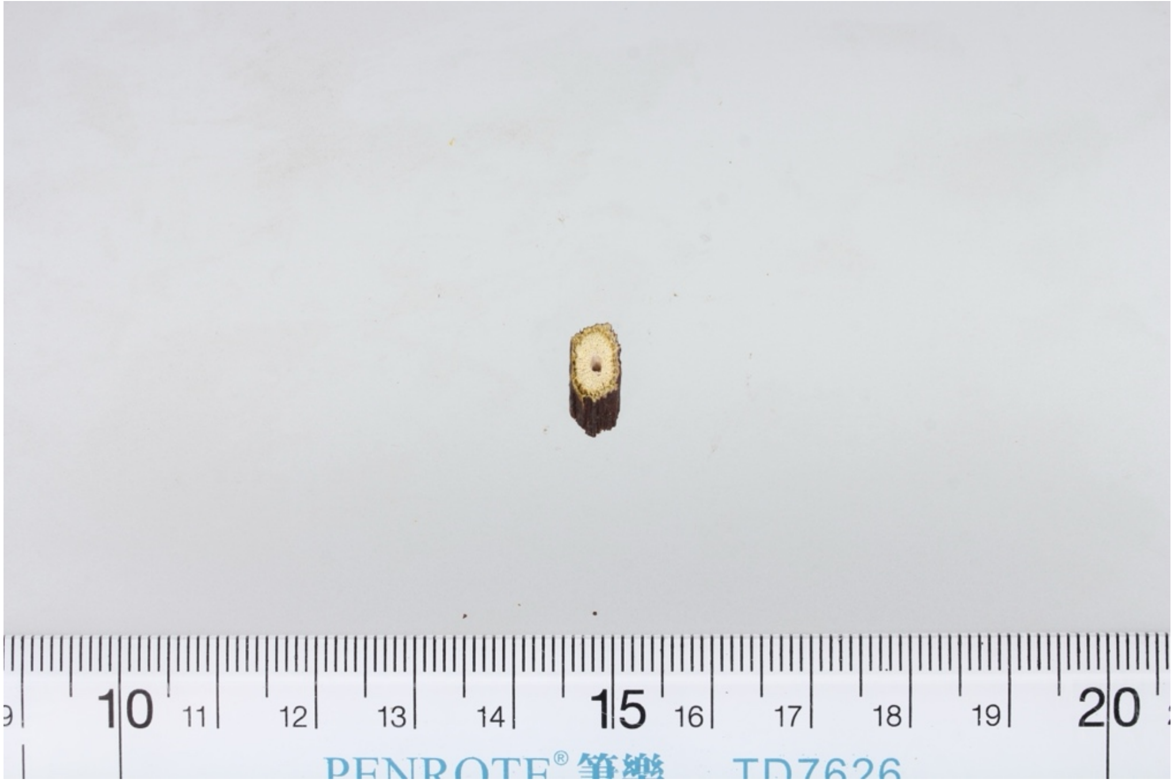

Figure 2. External morphology of stems and rhizomes, as well as adventitious roots from the same original *P. multiflorus* var. *hypoleuca* (PMH) plant.

(A) A crude form of PMH consists of stems (PMHC1, PMHC2, and PMHC3), rhizomes (PMH-R), and adventitious roots (PMH-A). Clearly, the roots of PMH are not enlarged like those of PM. This presents a significant visual difference between PM and PMH. (B) PMHC1, PMHC2, and PMHC3 samples are categorized on the basis of their diameter: 2.4–2.7 mm (PMHC1), 6.5–10.0 mm (PMHC2), and 21.0–30.0 mm (PMHC3). (C) The PMH-R and PMH-A samples enlarge image.

(A)

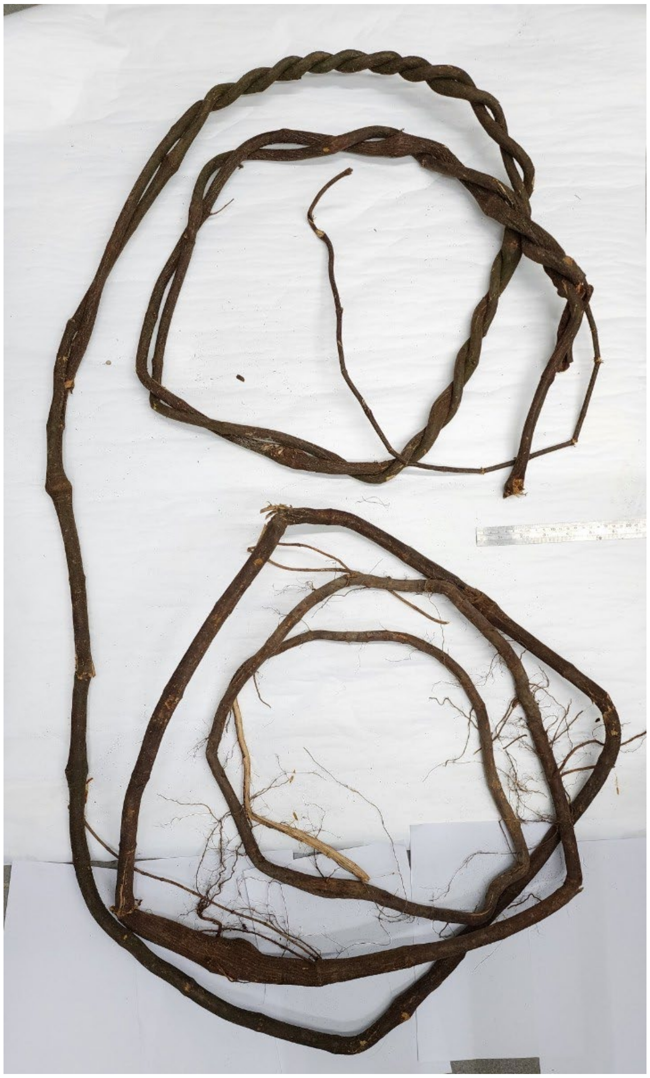

(B)

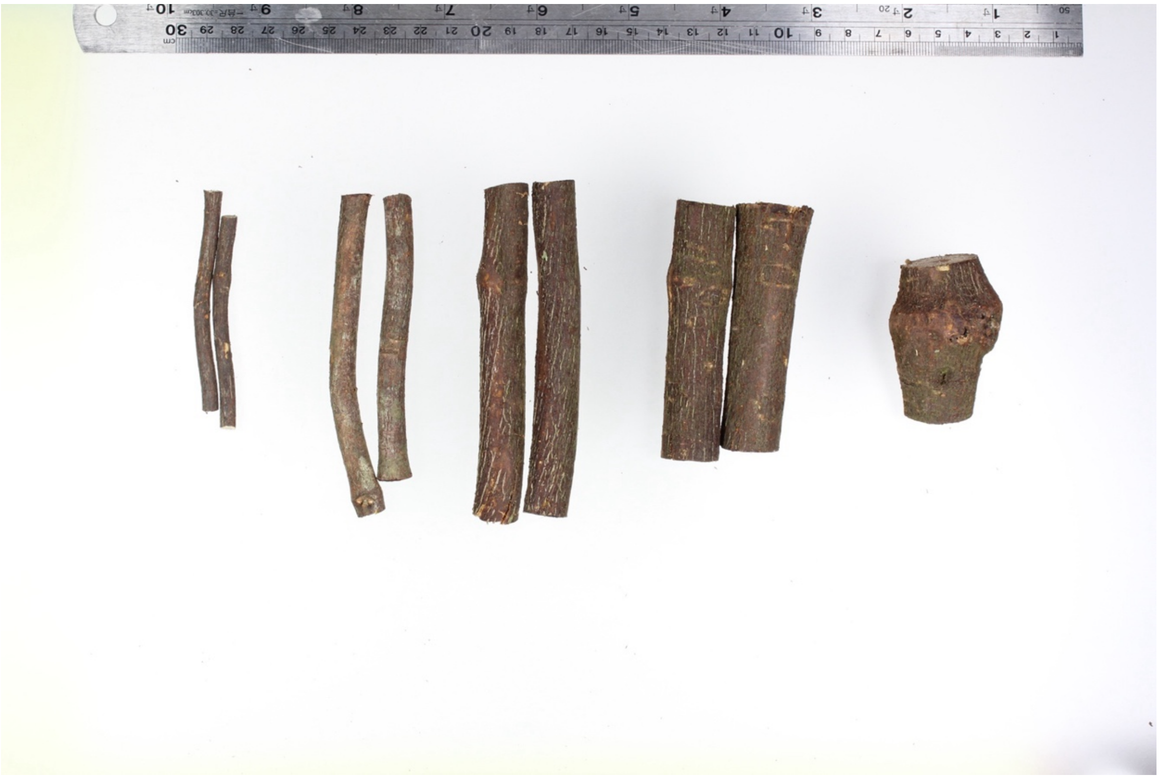

(C)

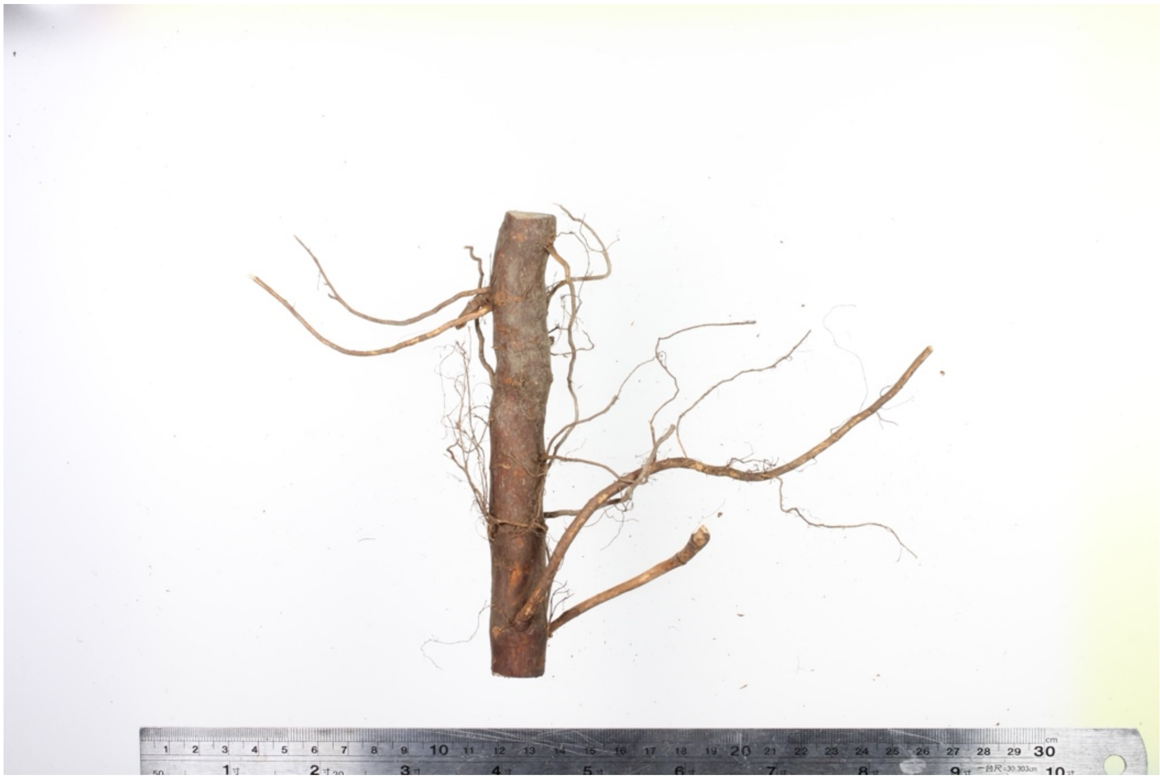

Figure 3. The representative view of cross-sectional view of the APMC, APMHC and APAC samples.

APMC1

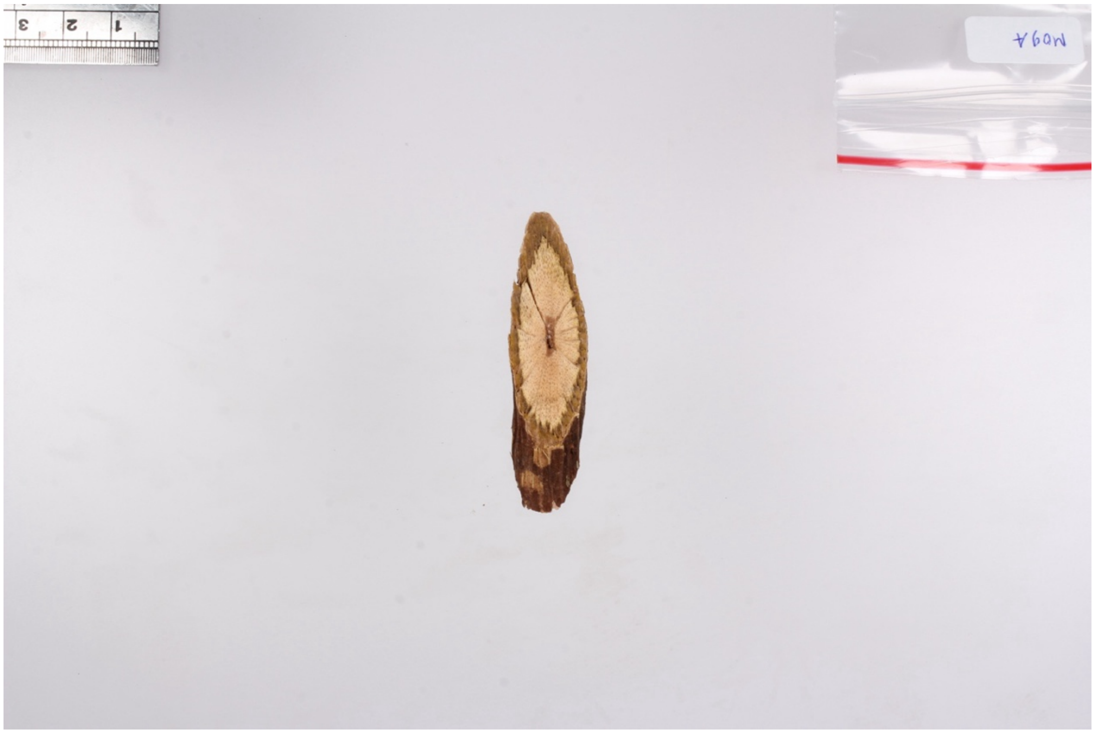

APMC2

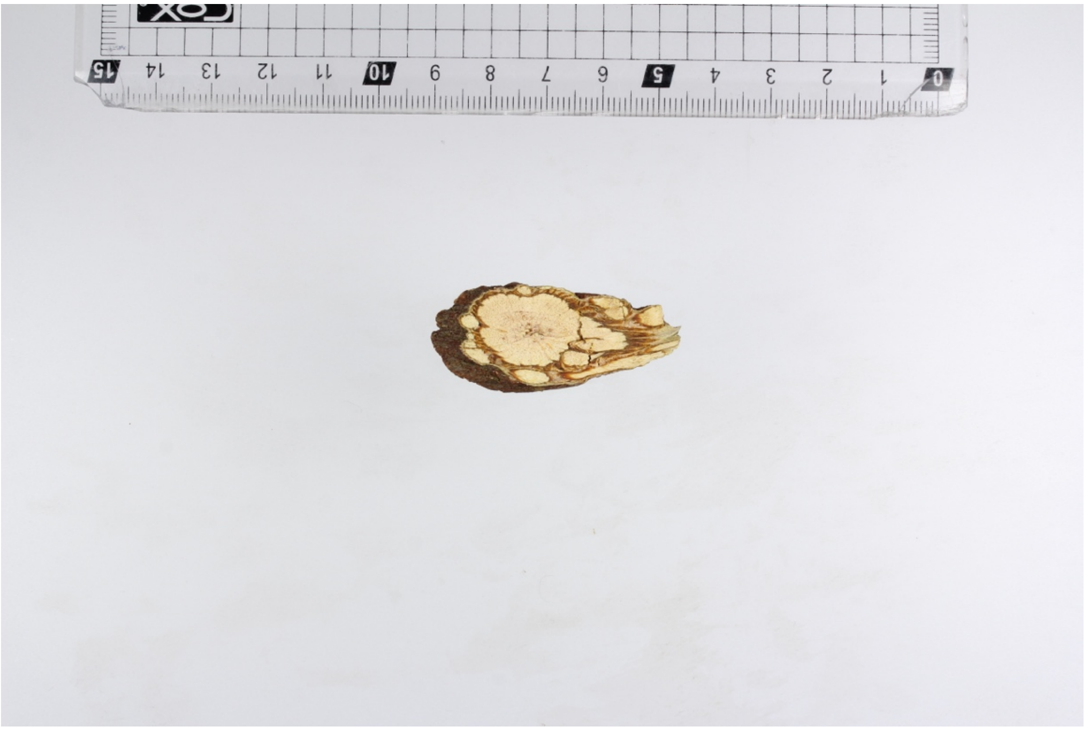

APMC3

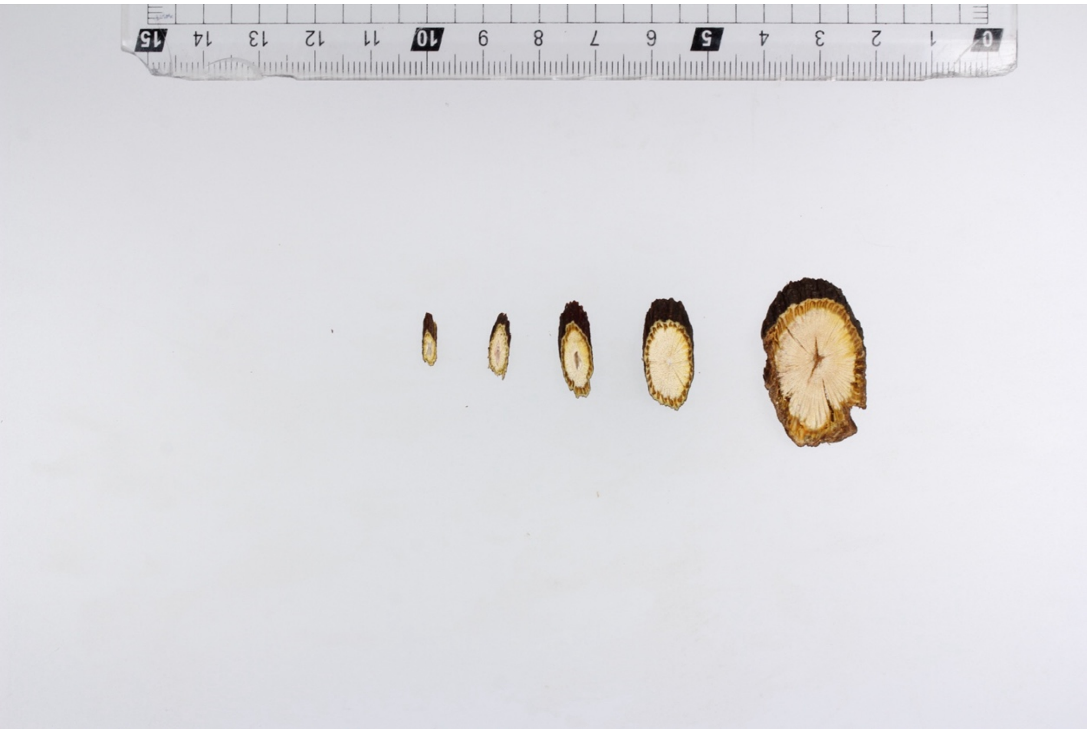

APMHC1

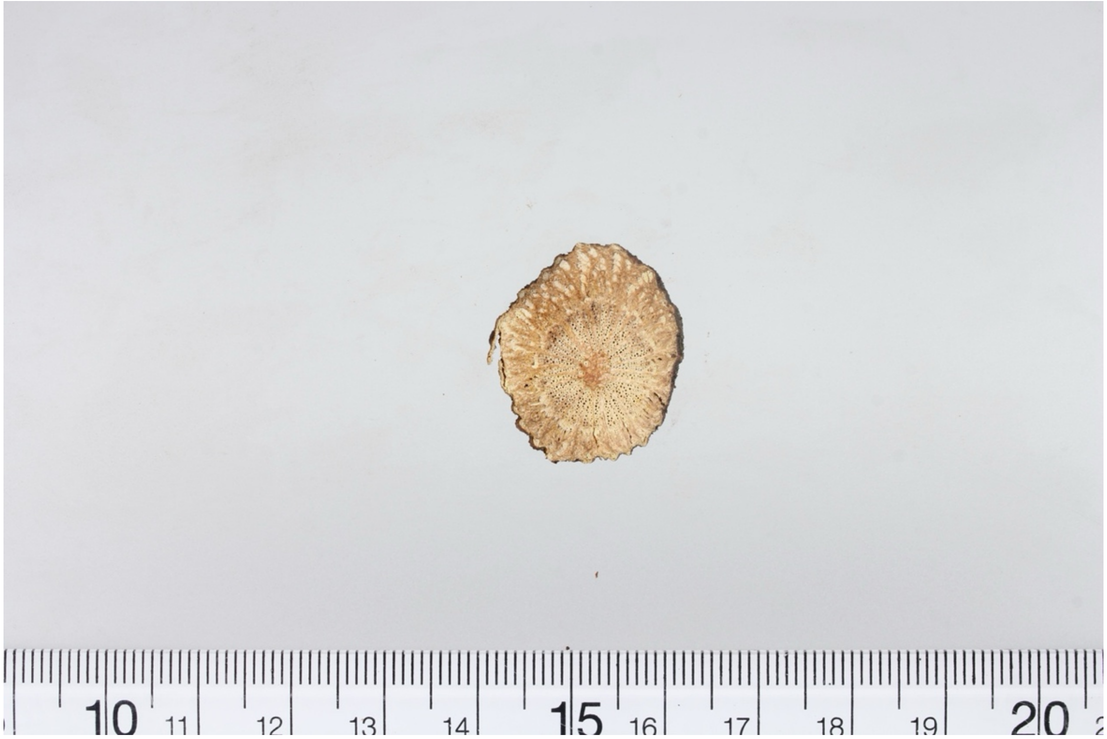

APMHC2

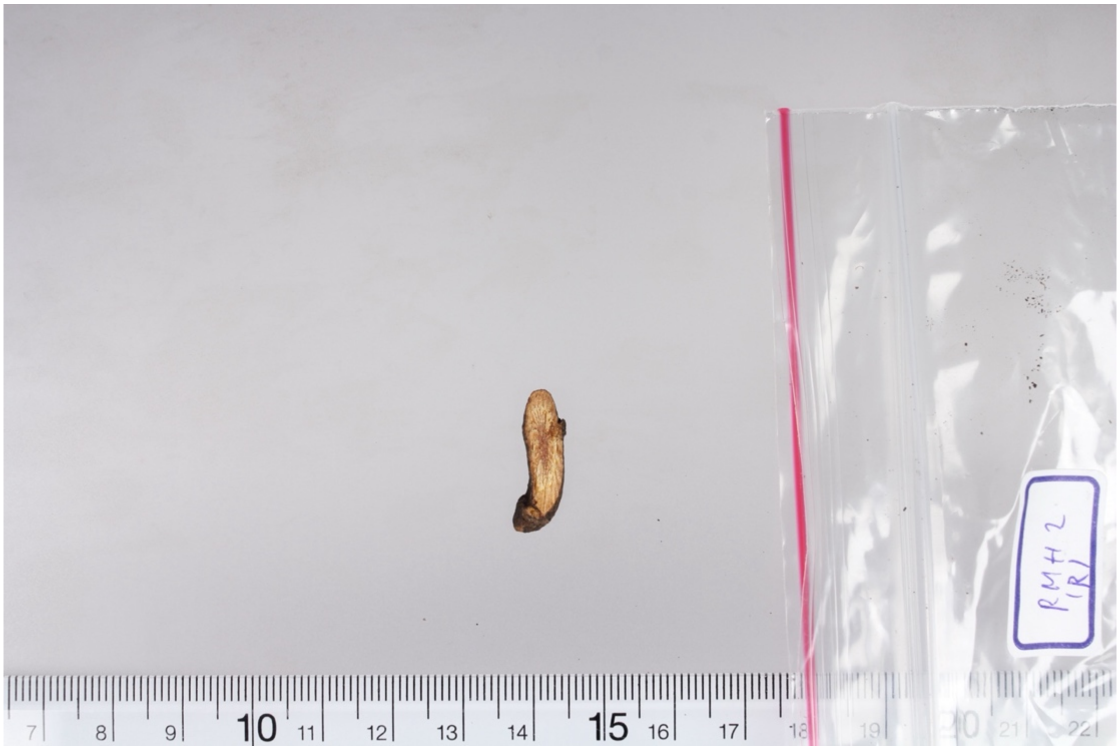

APMHC3

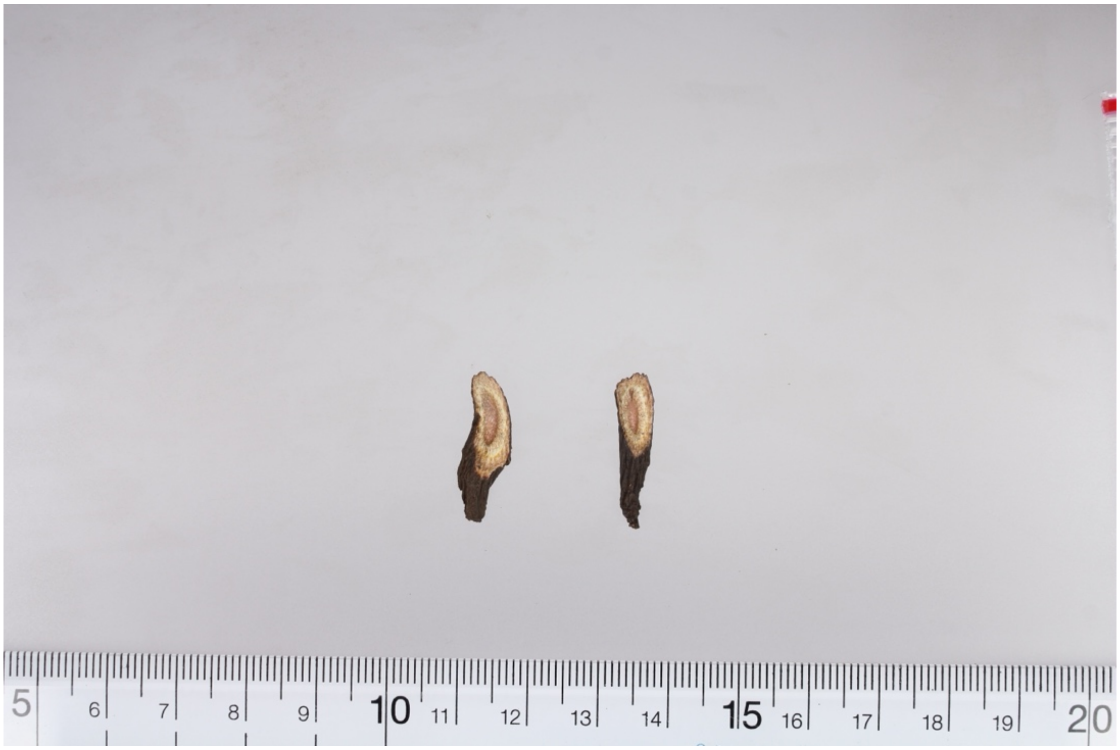

APAC1

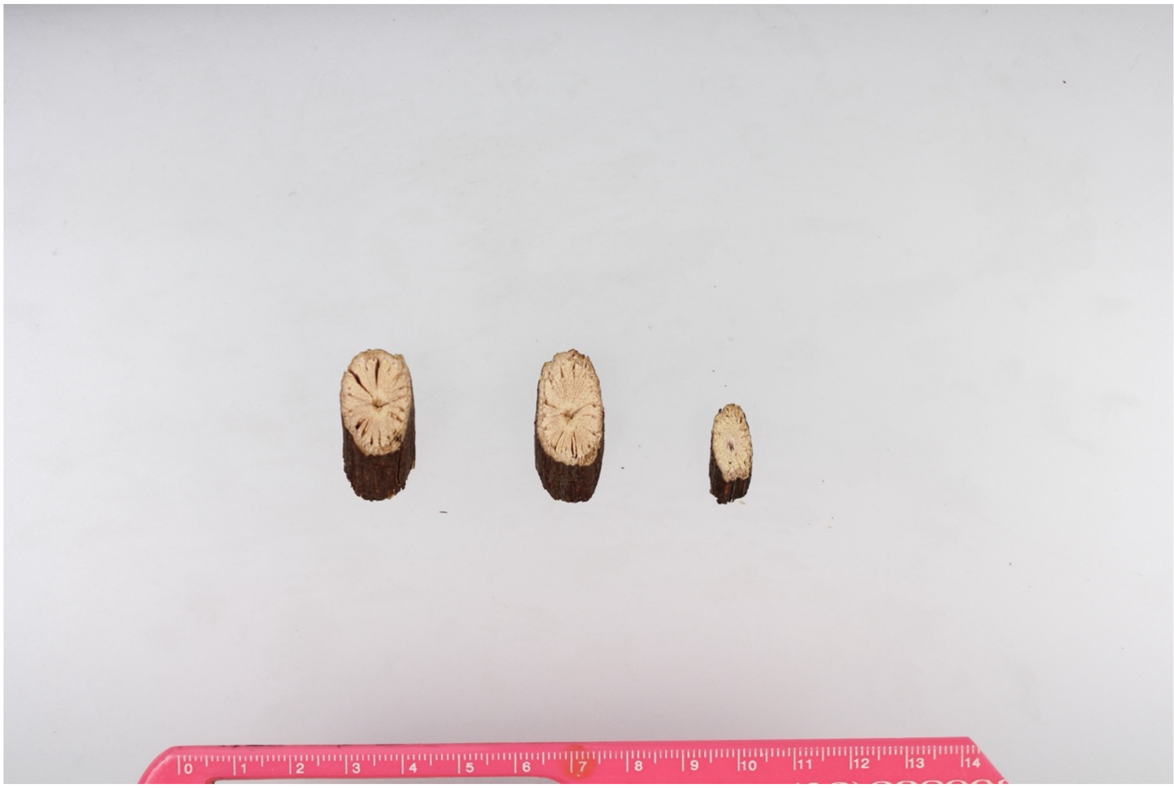

APAC2

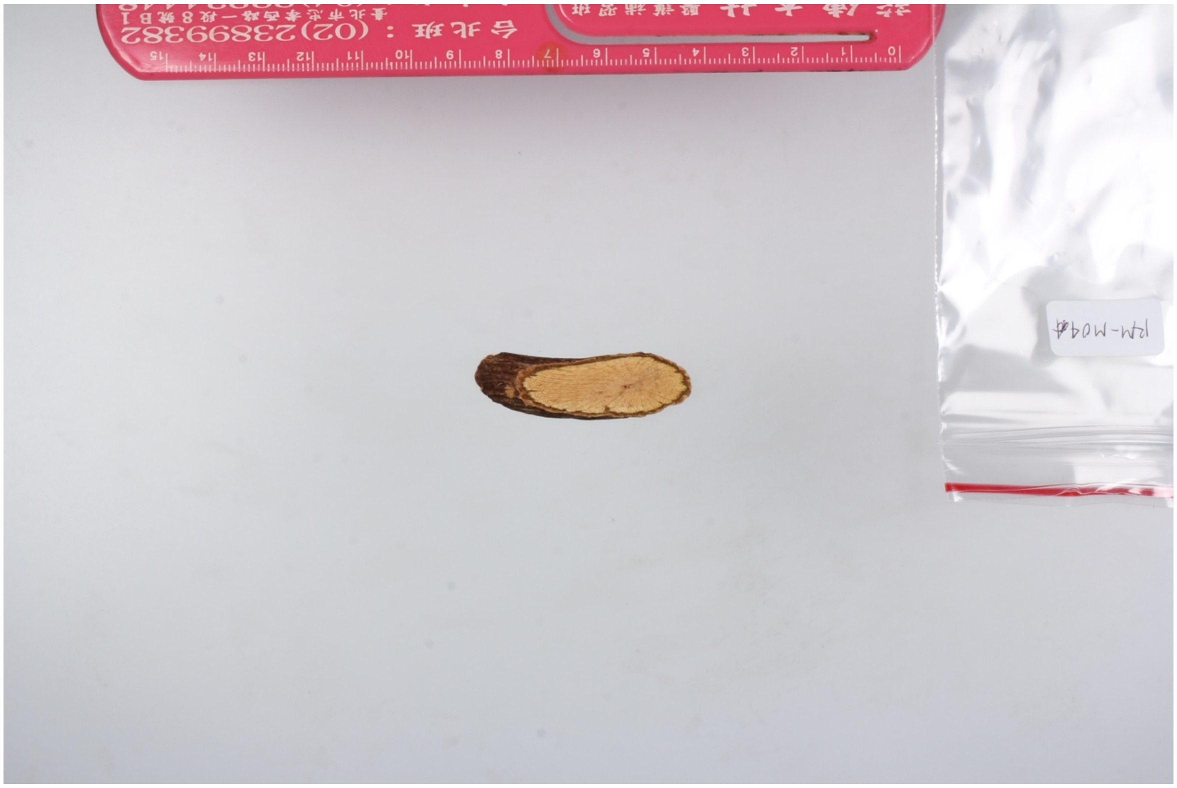

APAC3

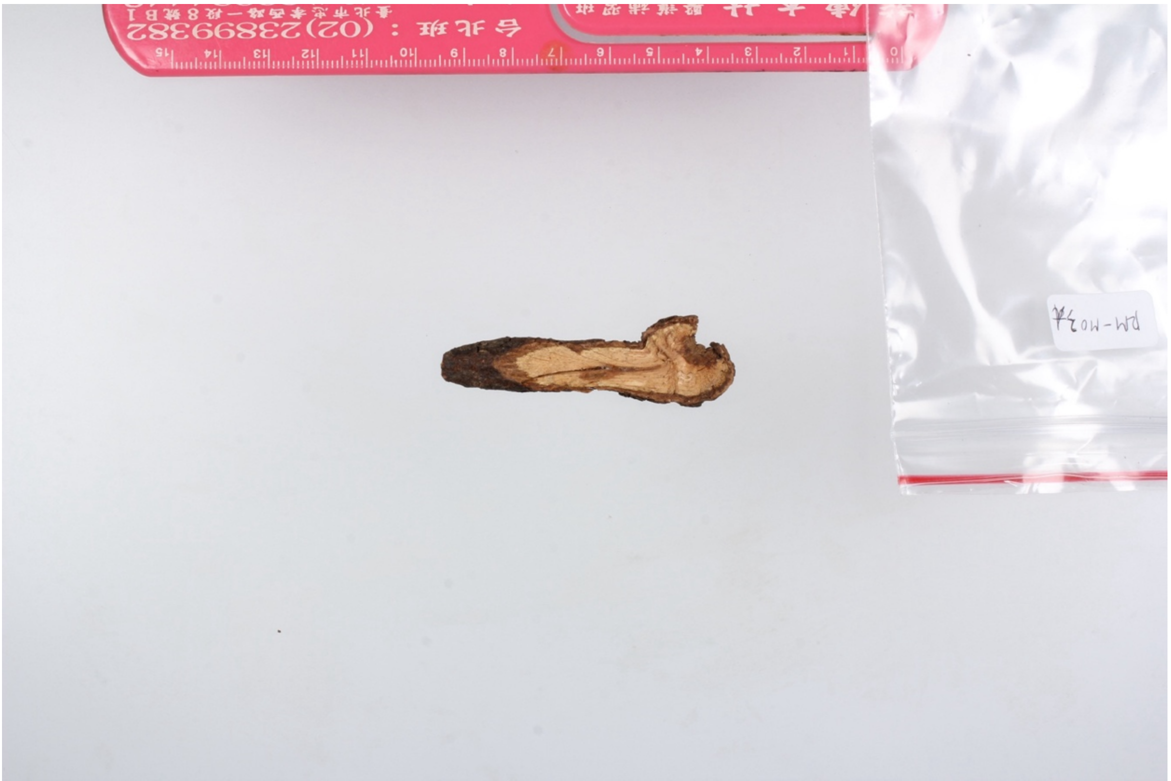

Supplement: Supplementary file 1 [file peerj-14-21561-s001.zip › peerj-130811-Raw_data_v2/Supplementary Data S1 Morphology original photos Figure 1-3.pdf]
