# Supplementary material for: Integrated chemical and genetic authentication of Polygoni Multiflori Caulis: Refuting diameter restrictions and exposing market adulteration
Source: PeerJ. 2026 Aug 3;14:e21561. doi: 10.7717/peerj.21561 (PMC13446225; doi:10.7717/peerj.21561)
Supplement: Supplementary file 1 [file peerj-14-21561-s001.zip › peerj-130811-Raw_data_v2/Supplementary Data S3 HPLC chromatogram of Figure 4.pdf]

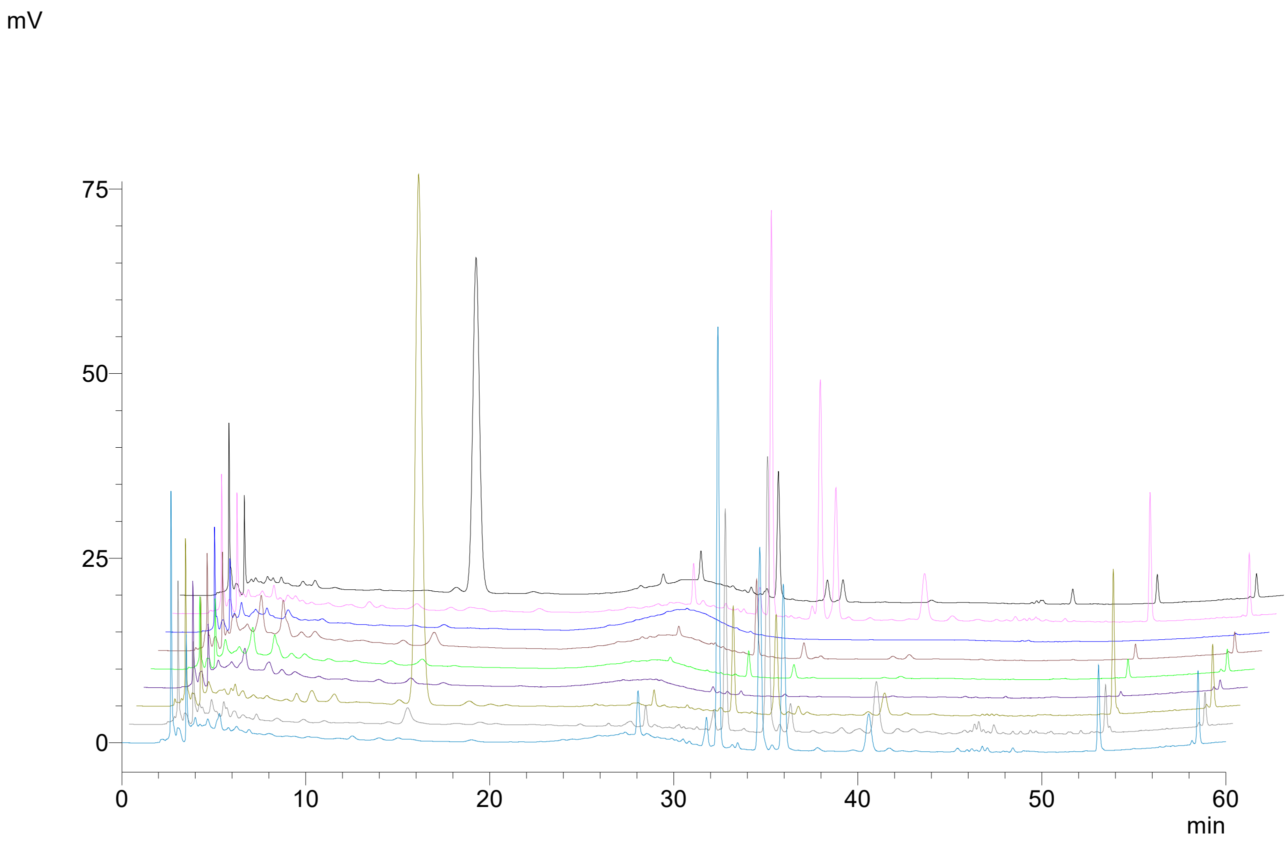
mV
75
50
25
0
0
10
20
30
40
50
60
min
